# Successes and Challenges in Implementation of Radon Control Activities in Iowa, 2010–2015

**DOI:** 10.5888/pcd13.150596

**Published:** 2016-04-14

**Authors:** Allison A. Bain, Anne L. Abbott, Laura L. Miller

**Affiliations:** Author Affiliations: Anne L. Abbott, Laura L. Miller, Center for Evaluation and Research, University of Iowa, Iowa City, Iowa.

## Abstract

**Background:**

Radon gas has recently become more prominent in discussions of lung cancer prevention nationally and in Iowa. A review in 2013 of cancer plans in the National Comprehensive Cancer Control Program found that 42% of cancer plans, including Iowa’s, had terminology on radon. Plans included awareness activities, home testing, remediation, policy, and policy evaluation.

**Community Context:**

Iowa has the highest average radon concentrations in the United States; 70% of homes have radon concentrations above the Environmental Protection Agency’s action levels. Radon control activities in Iowa are led by the Iowa Cancer Consortium, the Iowa Department of Public Health, and the Iowa Radon Coalition.

**Methods:**

A collaborative approach was used to increase levels of awareness, testing, and (if necessary) mitigation, and to introduce a comprehensive radon control policy in Iowa by engaging partners and stakeholders across the state.

**Outcome:**

The multipronged approach and collaborative work in Iowa appears to have been successful in increasing awareness: the number of radon tests completed in Iowa increased by 20% from 19,600 in 2009 to 23,500 in 2014, and the number of mitigations completed by certified mitigators increased by 108% from 2,600 to more than 5,400.

**Interpretation:**

Through collaboration, Iowa communities are engaged in activities that led to increases in awareness, testing, mitigation, and policy. States interested in establishing a similar program should consider a multipronged approach involving multiple entities and stakeholders with different interests and abilities. Improvements in data collection and analysis are necessary to assess impact.

## Background

Radon is a naturally occurring, radioactive, colorless, odorless gas produced by the decay of uranium. One route of radon production is through the soil. Radon in soil poses a health risk when the gas rises and enters buildings through cracks, joints, service pipes, and sump pits and accumulates indoors without having an exit. The Environmental Protection Agency (EPA) recommends that radon remediation take place when indoor levels reach 4 picocuries per liter (pCi/L) (1). As radon levels increase, health risks increase (2). Lung cancer is the leading cause of cancer death in the United States (3). Radon is the leading cause of lung cancer among nonsmokers and the second leading cause of lung cancer overall. An estimated 21,000 deaths occur nationwide each year as a result of radon exposure (4), making exposure prevention a priority issue.

A review published in 2013 of the National Comprehensive Cancer Control Program’s (NCCCP’s) plans found that only 42% of those plans had terminology on radon. Plans that did refer to radon were most likely to focus on awareness activities and included such topics as home testing, remediation, policy, and policy evaluation. The study concluded that NCCCP-funded programs should consider collaborating with other organizations to leverage resources used in identifying or controlling radon exposure and, when cancer plans are updated, should consider the following radon-related activities to prevent lung cancer (5): improve awareness of radon, improve home testing, promote remediation of homes with high levels of radon, support radon policy activities, and evaluate existing radon policy.

Iowa’s cancer plan is important for prioritizing radon-related activities in the state. The plan suggests the following activities: education, advocacy for comprehensive legislation, testing during real estate transactions, and financial assistance for radon mitigation (6). The plan is written, coordinated, and evaluated by the state’s cancer coalition, the Iowa Cancer Consortium (ICC), which is a 501(c)(3) nonprofit organization of more than 300 members who collaborate across the state.

The overall objective of Iowa’s radon interventions since 2010 has been to reduce the incidence of lung cancer in the state by reducing radon exposure through a collaborative approach. The creation of a statewide coalition and increasing community engagement activities improved awareness, increased radon testing, promoted remediation of homes when necessary, and instituted policy to increase radon testing and mitigation. These strategies for meeting the overall objective align with the recommendations of the review of NCCCP cancer plans (5).

Although interventions in Iowa began in 2010 before publication of the review in 2013, the review’s framework applies to interventions in Iowa since 2010 and is useful for highlighting broad success in the state. This article describes community engagement in Iowa to increase radon awareness, testing, and mitigation.

## Community Context

Iowa has the highest average concentrations of indoor radon in the United States; 70% of homes have radon concentrations greater than 4 pCi/L; this level is more than 6 times the national average and is due to soil composition (7). The entire state of Iowa is classified at the highest potential for radon exposure by the EPA; thus, all Iowans are at risk. These factors create a scenario that necessitates radon exposure prevention activities to reach Iowa’s 3.1 million residents (8).

Programs to address radon in Iowa are coordinated and radon stakeholders are mobilized by the Iowa Radon Coalition (IRC). (Materials created by the coalition are available at www.breathingeasier.info.) The IRC is made up of nearly 100 members who work on radon-related initiatives or are interested in increasing radon awareness. Coalition members include people and groups from various sectors: public health, nonprofit organizations, industry, health care, research, and academia (Figure). The IRC is facilitated by the ICC, and many IRC interventions are initiated and funded by the ICC. The ICC offers yearly community grants for radon awareness and testing projects. One radon coalition member group responsible for mobilizing communities across the state is lung cancer survivors. Survivors share their stories and the life-changing effects of radon exposure at community events and webinars and during legislative advocacy efforts.

**Figure:**
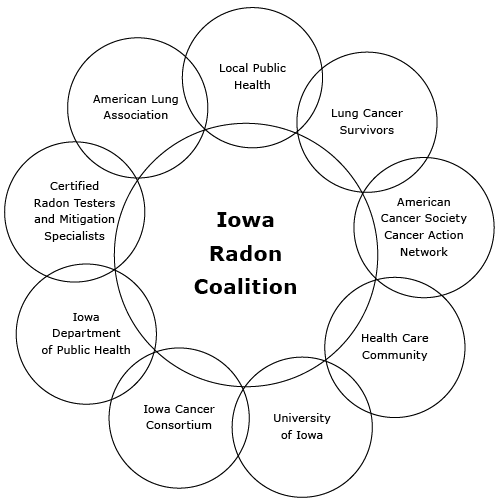
Members of the Iowa Radon Coalition.

The risk posed by radon exposure in Iowa makes the creation and promotion of radon policy a worthwhile endeavor. All lobbying activities are undertaken by the IRC. Since 2010, IRC members have advocated for testing and mitigation, when necessary, in Iowa’s schools. In 2014, these sustained lobbying efforts led to the passage of An Act Relating to Radon Control in Schools (9), which initiated the creation and administration of a survey for all public and private schools in Iowa. The results of this survey demonstrated that 82% of public and nonpublic schools had not implemented a radon testing and mitigation plan before December 1, 2014. Of those schools, 59% of public schools and 63% of private schools had no plans to create a testing and mitigation plan (10). The survey results underscored the need to continue advocating for a mandate on radon testing, a mitigation plan, and funds to assist schools.

Before the IRC’s formation in 2010, some radon-related policies were already in place. Since 1988, Iowa has required radon professionals to be licensed for radon testing and mitigation by the Iowa Department of Public Health (IDPH) (11). Additionally, although testing is not required for most entities in the state, testing has been required for daycare and preschool facilities since 1997 (12). In 2009, Iowa established a requirement for home sellers to inform home buyers of previous radon testing during real estate transactions, and realtors are required to share the Radon Fact Sheet during a real estate transaction (13). In 2015, Iowa amended disclosure language to include disclosure of results of a radon test, regardless of the level, to a potential buyer during a real estate transaction. Previously, only the seller received the results (13). These policy advances laid an important foundation for the current policy work by IRC members.

In 2013, the IRC first proposed creating policy to mandate that all new homes be built using radon-resistant new construction (RRNC) practices. RRNC practices are a series of techniques applied during the building process to prevent radon gas from entering the home, and the practices include having a passive mitigation system during construction (14). Although Iowa does not have a statewide policy on RRNC, RRNC codes have been adopted in 8 counties and 8 city jurisdictions (15). The decreased expense for the homeowner (compared with traditional mitigation) to activate the passive system makes the technique appealing to those considering or currently building homes. A statewide RRNC policy would help reduce one of the major barriers to mitigation — high costs — and potentially save each homeowner $500 to $1,500.

The IDPH is a member of the coalition. IDPH’s contributions occur through the Iowa Radon Program which tracks data on testing, mitigation, and certified mitigators. This program is coordinated by the Bureau of Radiological Health and the Bureau of Environmental Health Services. The program also provides national updates to the IRC. The Comprehensive Cancer Control Program in IDPH’s Bureau of Chronic Disease Prevention and Management provides oversight of radon activities implemented by the ICC. 

## Methods

When an intervention opportunity is initiated by the IRC, existing partners and coalition members are recruited. Many interventions are initiated by the ICC, and partners assist with developing materials or programs and then implementing programs in their community. Interventions are often modified to meet the needs of a target population. The partners’ community presence is leveraged to reach diverse populations across the state. Additional partners are recruited to address special needs related to awareness or testing, including targeted outreach to health care professionals, lobbying for policy, and outreach to particular populations or geographic areas. Partners are responsible for evaluating interventions and sharing outcomes of implementation.

Information is collected qualitatively, based on experiences, responses, and surveys, and quantitatively, by collecting data on radon levels, testing, and mitigation. Metrics used to track programs include data from pretests and posttests on knowledge during educational sessions and events. In addition, contact information for participants is collected when test kits are distributed during events so participants can be contacted by telephone or email if tests are not completed. Program evaluation data, including data on successes, barriers encountered, and reach, are also collected. Program outcomes are shared at meetings and through electronic communications with partners. Stories of successful initiatives are used to gain support and encourage partners to replicate activities in other areas of the state. Several examples of interventions are highlighted below.

### Improve awareness

A multifaceted communications effort was implemented to increase awareness of radon. Awareness activities occur during the entire year, but they increase during November’s Lung Cancer Awareness Month and January’s Radon Action Month. Advocates work with the governor’s office to declare each January Radon Action Month, giving radon a statewide profile, and to help increase awareness among Iowa’s legislators. Awareness activities include the creation of original content, material distribution, and media engagement. Original content included small media and social media. Social media messages are developed by the ICC and integrated into the social media accounts of partners. Among the most promising original content are 1) a brochure with Iowa-specific information, “Radon & You: What you need to know to protect you and your family” and 2) “Breathing Easier,” a YouTube video with 12-minute and 23-minute versions targeted to health care professionals (www.breathingeasier.info) (16). The brochure was developed by the IRC and the American Lung Association in English and Spanish, and it is available at no cost to the public. Partners distribute this brochure across the state as does the Iowa Radon Hotline, an 800-number that receives 1,400 calls per year from Iowans requesting information about radon. The hotline is funded by the Iowa Radon Program and staffed by the American Lung Association.

The IRC also used earned media to increase awareness. Earned media allows organizations to promote events, activities, and stories without a fee. In January 2015, media outreach activities included developing and distributing media packets with radon information, promoting local radon-related stories, and disseminating contact information for radon experts and media outlets across the state. Twenty-four radon–related news stories were published in January 2015.

### Increase radon testing and promote remediation of high radon levels

The importance of testing for and remediating high radon levels is included in Iowa’s radon awareness messaging; however, strategies to increase testing and remediation, when necessary, were also implemented. For testing, community-based projects and events that couple educational materials with on-site test-kit distribution is a promising practice. Participants are required to complete a form when receiving a kit and receive follow-up by telephone or email if their test has not been completed. The ICC provides funding for such events; the ICC supported nearly 40 individuals or organizations in implementing projects, some of which consisted of multiple events. The reach of these events varied from 10 to 500 people. Well publicized events with multiple partners including public health and health care partners had the highest reach.

Once testing is complete, the major barrier to mitigation is cost: approximately $1,200. With that in mind, the IRC and the ICC worked to find financial assistance programs for mitigation. The IRC worked with the Iowa Bankers Association to publish a newsletter article in January 2014 recommending that member banks offer unsecured low-interest loans for mitigation. To ensure that those who are not eligible for or unable to repay loans have mitigation options, the ICC and its partners disseminated information about assistance provided by local organizations. The ICC also provided services to communities with low socioeconomic status. For example, the ICC, from October 2012 through September 2014, in partnership with Polk County’s Healthy Homes Program, supplemented funding through the Radon Free Homes Initiative to test and mitigate homes at no cost to the homeowner through a social justice grant from the EPA. Additionally, some ICC-funded projects, such as the Northwest Iowa project, engaged in policy-, system-, and environmental-change efforts that addressed mitigation barriers. In the Northwest Iowa project, the grantee met with local banks to offer low-interest loans for home mitigation and 2 local contractors became certified mitigators in response to the project (17).

### Support radon policy activities and evaluate existing radon policy

The IRC supports and advocates for comprehensive radon legislation, including radon testing and mitigation in schools, financial assistance for mitigation, and RRNC. Model policy includes all of these elements. The diverse membership of the IRC empowers members who can lobby for radon-related legislation and encourages education and advocacy efforts among members who are prohibited from lobbying. IRC members, including the American Cancer Society Cancer Action Network, cancer survivors, and the Iowa Medical Society, are involved in lobbying for statewide radon policy. Advocacy activities include workshops to educate legislators about radon, an educational mailing to legislators, and one-on-one education. No formal evaluation of advocacy efforts with legislators was completed, however; anecdotal evidence from members of the coalition who work closely with legislators points to an increase in radon awareness.

## Outcome

Community engagement on the issue of radon exposure has improved because of these collaborative efforts. Before 2010, awareness messaging was limited because communication among organizations addressing radon was not coordinated. The IRC has helped coordinate awareness efforts, increase radon testing and mitigation, and focus policy efforts.

The collaboration of partners since 2010 led to an increase in the number of messages reaching Iowans and created a unified message about the dangers radon poses and what can be done to reduce risk. Since 2010, the IRC distributed more than 30,000 “Radon and You” brochures. As of December 2015, the 12-minute radon video, created by the University of Iowa College of Public Health and IRC members, had nearly 7,000 views, and the 23-minute video had more than 2,400 views. November’s Lung Cancer Awareness Month and January’s Radon Action Month were promoted on social media starting in 2013. One Facebook post, which featured a radon-induced lung cancer survivor, for Radon Awareness Month in 2014 had 1,000 unique views, 55 post clicks, and 44 likes, shares, or comments. Media outreach efforts resulted in 24 radon-related stories on television and radio and in newspapers during Radon Action Month in 2015.

Funding provided by the ICC for radon-related initiatives has totaled more than $57,500 since 2010. Since then, 9 projects mobilized Iowans to provide education; test for radon; and create policy, systems, and environmental changes. In one Northwest Iowa community, the ICC funded a family physician to hold community education sessions, which more than 400 people attended and which resulted in 378 homes being tested for radon; more than 85% of homes had test results greater than 4.0 pCi/L (14).

Radon testing and mitigation has increased since the collaborative work began in Iowa. In 2009, before implementation of collaborative radon efforts, the number of radon tests completed in Iowa was 19,600. In 2014, after implementation, nearly 23,500 radon tests were completed — an increase of 20%. Evidence suggests that efforts to promote mitigation were successful. In 2009, the number of mitigations completed by certified mitigators was 2,600. In 2014, that number increased to more than 5,400, a nearly 108% increase (18). The number of certified mitigation specialists who perform remediations in the state increased from 54 in 2009 to 76 in 2014 (18), which suggests that demand is increasing. Although these increases do not address the full 1.3 million residences in Iowa (8), the increases are positive.

## Interpretation

Iowa communities are engaged in the issue of radon exposure through collaborative efforts, and this engagement has led to improvements in awareness, testing, mitigation, and policy. States interested in establishing a similar program should consider a multipronged approach involving multiple entities and stakeholders with different interests and abilities (Table).

**Table:** Radon Activities by Iowa Cancer Consortium, Iowa National Comprehensive Cancer Control Program, and Iowa Radon Coalition, 2010 to 2016

Radon-Related Activity Area (5)	Examples of Activities
Improve awareness of radon	Created Iowa-specific educational materials, including a brochure (http://canceriowa.org/ICC/files/c7/c774551a-687e-4a3e-8561-367d0a780737.pdf) and educational video (www.youtube.com/watch?v=DXn5s7-QCJY)
	Operate Iowa Radon Hotline through the American Lung Association
	Educated health care professionals on radon
	Direct outreach to various organizations, including worksites, real estate groups, banks, and garden clubs
	Created and coordinated social media messages on Facebook and Twitter
	Partnered with local television stations and newspapers
Improve home testing	Distributed test kits at large and small local and statewide events
	Educate about testing methods with instructions and demonstrations
	Attended conferences and events as speakers and vendors
Promote remediation of homes with high levels of radon	Supported financial assistance programs for mitigation
	Worked with Iowa Bankers Association on an article on banks that offer low-interest loans for home mitigation
	Developed a financial-resources document for Polk County that highlights programs that include radon testing and mitigation as part of services
Support radon policy activities	Involved in state and federal legislation on school testing and mitigation, radon-resistant new construction, and funding for mitigation and education
	Educated state legislators about radon and provided test kits
	Work with the Iowa governor’s office to recognize Radon Action Month
Evaluate existing radon policy	Evaluate state radon policy annually

One challenge in evaluating the success of Iowa’s radon efforts is the collection and analysis of state radon data. Currently, IDPH receives data from test-kit laboratories, which report the information provided by the homeowner or tester, and compiles data on the number of tests completed. However, the absolute number of test kits completed is often misleading because it does not reflect the number of homes tested. The number of test kits used to test a home depends on square footage and the personal choice of the tester. Additionally, these data are not readily available to the public or to advocates of radon testing; this lack of availability presents concerns not only for program evaluation but also for members of the general public who are interested in learning about radon levels in their community.

Data on the public’s level of radon awareness are also lacking. The level of activity and engagement by entities such as the ICC, IRC, and IDPH suggests that the public’s level of awareness of radon increased, but no data confirm this supposition. Some states use surveys such as the Behavioral Risk Factor Surveillance Survey (BRFSS) to capture these data, but Iowa does not.

In creating best practices for radon activities in other jurisdictions, it is important to note that Iowa’s success statewide is not well evaluated, in part because of the challenges in collecting test data. Statewide test and mitigation data are currently collected and stored in a way that is not easy to access or analyze. Currently, the ICC collects data at the individual and project level to overcome the limitations of statewide data collection. Iowa’s experiences make a viable case for collecting data on each radon-related activity.

The challenges for data collection and analysis do not diminish the gains made in Iowa to date, however, and IDPH is currently working to improve the data collection system to make data more accessible to advocates for radon testing and the general public. The improvements in data collection and availability will be valuable for future campaigns and for determining where collaborative efforts are most effective. 

Overall, the multipronged approach and collaboration appears to be successful in increasing awareness and testing of radon in Iowa. Policy change will be necessary to create large-scale changes in the state and will continue to be pursued by the various agencies involved in radon control activities. Improvements in data collection and analysis are necessary to accurately assess the impact of the multipronged approach and collaboration.
